# BCL3 Expression Is a Potential Prognostic and Predictive Biomarker in Acute Myeloid Leukemia of FAB Subtype M2

**DOI:** 10.1007/s12253-018-0476-7

**Published:** 2018-10-25

**Authors:** Yuna Niu, Xue Yang, Yifei Chen, Linbo Zhang, Xinyue Jin, Youjing Tang, Li Li, Lu Yu, Yilin Guo, Hui Wang

**Affiliations:** 1 Henan Collaborative Innovation Center of Molecular Diagnosis and Laboratory Medicine, School of Laboratory Medicine, Xinxiang Medical University, Xinxiang, Henan 453003 People’s Republic of China; 2 Henan Key Laboratory of Immunology and Targeted Therapy, Xinxiang Medical University, Xinxiang, Henan 453003 People’s Republic of China; 3 Department of Laboratory Medicine, the Third Affiliated Hospital Of Xinxiang Medical University, Xinxiang, Henan 453003 People’s Republic of China; 4 Laboratory of Hematology, the First Affiliated Hospital of Xinxiang Medical University, Weihui, Henan 453003 People’s Republic of China

**Keywords:** BCL3, Expression, Prognosis, Acute myeloid leukemia

## Abstract

Although the implication of *BCL3* has been disclosed in human chronic lymphocytic leukemia as well as other solid tumors, the diagnostic and prognostic of *BCL3* expression in acute myeloid leukemia (AML) remains largely unclear. In this study, we isolated total RNA from bone marrow mononuclear cells collected from 101 de novo AML patients and 27 healthy donors. After reverse transcription, quantitative real-time PCR was performed to detect *BCL3* expression level. *BCL3* mRNA level was significantly down-regulated in BMMCs of AML patients compared with healthy controls (*P* = 0.0015). *BCL3* was showed a higher level in AML patients with poor-risk karyotypes than that of in patients with favorable/intermediate-risk karyotypes (*P* = 0.014). ROC analysis demonstrated that *BCL3* could effectively differentiate AML patients from normal controls. Among the French-American-British (FAB) subtypes, the frequency of low *BCL3* expression in M2 subtypes is significantly higher than that of in the other subtypes M1/M4/M5/M6/M7 (*P* = 0.006), and mildly lower in myelomonocytic/monocytic subtypes M4/M5 (*P* = 0.064) than those in M1/M2/M6/M7 subtypes. Chromosome analysis revealed that *BCL3*^low^ patients had a remarkably higher frequency of t (8;21) abnormality (*P* = 0.0047) and lower frequency of normal karyotype (*P* = 0.0059) than *BCL3*^high^ patients. *BCL3*^high^ patients showed a significantly higher frequency of FLT3-ITD mutation (*P* = 0.028) and lower frequency of C-Kit mutation (*P* = 0.0232) than *BCL3*^low^ patients. Although there were no significant differences in complete remission and overall survival between BCL3^low^ and BCL3^high^ groups, patients with high BCL3 expression markedly shorter overall survival (OS, *P* = 0.049), relapse-free survival (RFS, *P* = 0.027) and disease-free survival (DFS, *P* = 0.042) in M2 AML than low BCL3 expression patients. Additionally, in AMLs of M2 subtype, high BCL3 expression patients had markedly lower complete remission (CR) rate (*P* = 0.0317) after the second induction treatment than patients with BCL3 low expression. Thus, these findings indicated that BCL3 appeared as a promising molecular biomarker of pediatric acute myeloid leukemia with unfavorable prognosis.

## Introduction

Acute myeloid leukemia (AML) is a cancer of the myeloid line of blood cells, which comprises approximately 15–20% of pediatric leukemia[1]. Although the cure rate has improved, treatments are associated with notable morbidity and mortality. About 60–70% of patients achieve complete remission after the induction chemotherapy, only 20–30% of patients achieve long-term disease-free survival (DFS) [2–4]. Each AML patients can be separated into distinct risk subgroup. The diagnostic classification of pediatric AML depends on the combination of morphology, cytochemistry, immunophenotyping and molecular genetics. Moreover, aberrant expression of cancer-related genes has been associated with the prognosis and treatment outcome of AML.

BCL3 gene was initially identified in B cell chronic lymphocytic leukemia (B-CLL) with chromosomal translation of t(14;19)(q32;q13), which encodes a nuclear protein that belongs to the IkB family of inhibitors of NFkB [5]. Nevertheless, latter research has shown that BCL3 rearrangement is recurrent in other hematological malignancies, such as T cell lymphoma, Burlitt-like lymphoma, Hodgkin lymphoma [6–8]. Disregulation of BCL3 without chromosomal translocation has been found to participate in progression in a variety of solid tumors, from breast cancer, nasopharyngeal carcinoma and renal-cell carcinoma to lung cancer [9–12]. Moreover, abnormal expression of BCL3 was associated with prognosis in patients with CLL, breast cancer, clear-cell renal-cell carcinoma and non-small-cell lung cancer [12–15]. Due to its overexpression in numerous types of tumors, as well as its extensive roles in promoting transformation in hematologic malignancies, BCL3 has been recognized as an oncogenic gene. However, up to now, little studies investigated BCL3 expression or its role in myeloid malignancies. The current study was aimed to investigate BCL3 expression and its clinical significance in pediatric patients with de novo AML.

## Materials and Methods

### Patients and Control Group

This original research study included 100 patients who had previously been diagnosed with AML at the Children’s Hospital Affiliated to Suzhou University as well as 27 normal controls. None of the patients had received any treatment before bone marrow (BM) sample collection. The diagnosis and classification of AML were based on the French-American-British (FAB) and World Health Organization (WHO) criteria. Complete remission (CR) was defined as that the bone marrow contained less than 5% blasts. Overall survival was defined as the period of time after patients were diagnosed with disease. Disease-free survival (DFS) was the length of time after treatment during which no disease is found. Details of clinical characteristics of all AML patients were summary in Table 1.

**Table 1:** Correlation between BCL3 expression and clinical parameters of 100 AML patients

Patients’ parameters	BCL3 expression		
	Low (*n* = 50)	High (n = 50)	*P* value
Sex, male/female	33/16	25/26	0.063
Median age, month, (range)	91.67 (9–149)	78 (7–166)	0.215
Median WBC, ×10^9^/L (range)	15.5 (0.42–275.9)	16.9 (1.05–300.55)	0.811
Median HGB, g/L (range)	75 (20–111)	77.67 (1.96–108)	0.947
Median PLT,×10^9^/L, (range)	41 (10–179)	66.33 (10–586)	0.017
BM blasts, % (range),	63 (15–95)	72.5 (12–98)	0.699
PB blasts, % (range)	56.8 (2–91)	41.33 (2–94)	0.201
FAB classification	*n* = 47	n = 50	
M1	1 (1%)	3 (3.1%)	0.338
M2	30 (30.9%)	18 (18.6%)	0.006
M4	7 (7.2%)	13 (13.4%)	0.177
M5	9 (9.3%)	13 (13.4%)	0.420
M4 + M5	16	26	0.064
M6	0 (0%)	1 (1%)	0.330
M7	0 (0%)	2 (2.1%)	0.166
Karyotypes	n = 50	n = 50	
Normal	4 (4%)	15 (15%)	0.0059
t(8;21)	23 (23%)	10 (10%)	0.0047
Inv(16)	5 (5%)	8 (8%)	0.3936
+ 8	0 (0%)	1 (1%)	0.3197
-5/5q	1 (1%)	0 (0%)	0.3101
-7/7q	0 (0%)	1 (1%)	0.3197
MLL rearrangement	4 (4%)	7 (7%)	0.3558
Complex	4 (4%)	2 (2%)	0.3860
Other	5 (5%)	4 (4%)	0.7037
No data	4 (4%)	3 (2%)	0.6752
Karyotype classification	*n* = 46	*n* = 51	0.278
Favorable	18	17	
Intermediate	24	22	
Unfavorable	7/	12	
Gene mutation	*n* = 49	n = 51	
No mutation (± )	14	8	0.1339
C-Kit (± )	9	2	0.0232
FLT3-ITD (± )	1	5	0.0486
CEBPA	0	2	0.1573
NPM1	1	0	0.3101
No data	24	34	0.0893
CR			
CR1 (CR/PR/NR)	30/12/7	22/21/8	0.156
CR2 (CR/PR/NR)	36/5/8	36/9/6	0.499

AML, acute myeloid leukemia; WB, white blood cells; HB, hemoglobin; PLT, Platelet; BM, bone marrow; PB, peripheral blood; FAB, French-American-British; CR, complete remission; PR, partial remission; NR, no remission

This research was approved by the institutional Ethics Committee of the Children’s Hospital Affiliated to Suzhou University. A written informed consent was also signed from each participant of the study.

### RNA Isolation and Reverse Transcription

BM mononuclear cells (BMMCs) were isolated by Lymphocyte Separation reagent (Dakewei, Shenzhen, China) mediated density gradient centrifugation according to the protocol. Total RNA was extracted using Trizol reagent (Invitrogen, Carlsbad, CA, USA), the concentration and purity of total RNA was assessed by using NanoDrop 2000 Spectrophotometer. Reverse transcription was performed to synthesize cDNA using the PrimeScript RT reagent kit (Takara, Dalian, China). The system of Reverse transcription was incubated for 60 min 42 °C, followed by 5 min at 95 °C.

### Real-Time Quantitative PCR

*BCL3* expression was examined by real-time quantitative PCR (Q-PCR) performed on a 7500 thermo cycler (Applied Biosystems, CA, USA). SYBR Premix Ex Taq II kit (Takara, Dalian, China) was used for Q-PCR reactions with the following primers, forward 5’ CGTGAACGCGCAAATGTACT 3′ and reverse GATGTCGATGACCCTGCGG. The Q-PCR reaction was carried out at 95 °C for 5 min, followed by 40 cycles at 95 °C for 10 s, 60 °C for 30 s, 72 °C for 30 s and 70 °C for 30 s to collect fluorescence. Relative *BCL3* expression levels were calculated by 2-^△△CT^ method against GAPDH reference gene.

### Statistical Analysis

Statistical analysis was performed using the SPSS 19.0 software package. Non-parametric Mann-Whitney U test was used to compare the distributions of *BCL3* expression value in AML patients and healthy controls. Person Chi-square analysis or Fisher exact test were carried out to compare the difference of categorical variables. We also assessed the diagnostic potential of *BCL3* expression by receiver operating characteristic (ROC) analysis. Thus, a ROC curve was built by plotting sensitivity versus 1-sepecicity, and the respective area under the curve (AUC) was analyzed by the Hanley and McNeil method. Kaplan-Meier overall survival (OS) analysis was performed, and differences between OS curves were evaluated using the Mantel-Cox (log-rank) test.

## Results

### BCL3 Expression in Controls and AML Patients

**Fig. 1:**
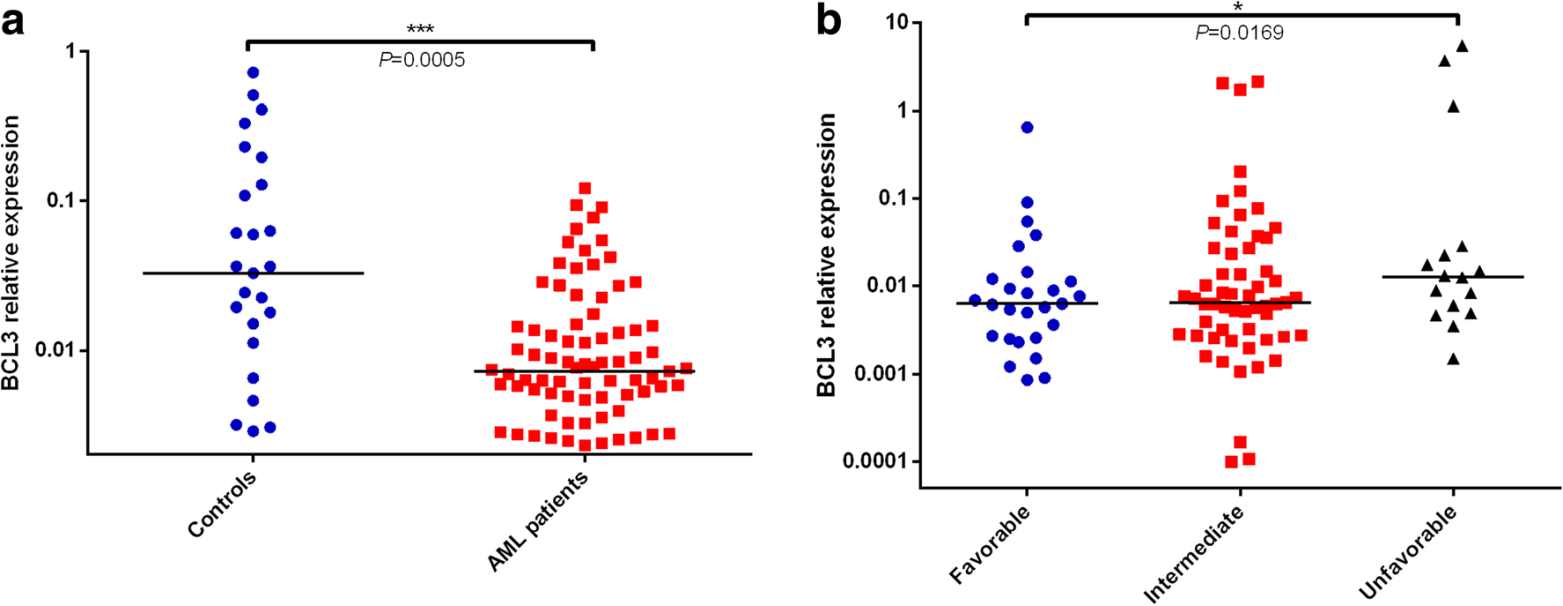
Relative expression level of *BCL3* in AML patients and controls. **a** The *BCL3* levels in AML patients were lower than those in controls (Mann-Whitney-U test). **b** The *BCL3* levels in AML patients with favorable-risk cytogenetics group (One-Way anova analysis). The distributions of the *BCL3* expression were presented with scatter plots. The median level of *BCL3* expression in each group was shown with horizontal line

Expression of *BCL3* in AML patients and healthy donors was detected by qRT-PCR. In contrast to controls (median 0.0032, range 1.25E5–0.0072), BCL3 (median 0.0071, range 0.00032–5.5277) *BCL3* mRNA levels were significantly decreased in AML patients (*p* < 0.001, Fig. 1a). However, AML patients in unfavorable-risk cytogenetics group had much higher *BCL3* expression than that in intermediate-risk or favorable-risk cytogenetics group (*p* < 0.05, Fig. 1b).

### Discriminative Capacity of BCL3 Expression in AML

**Fig. 2:**
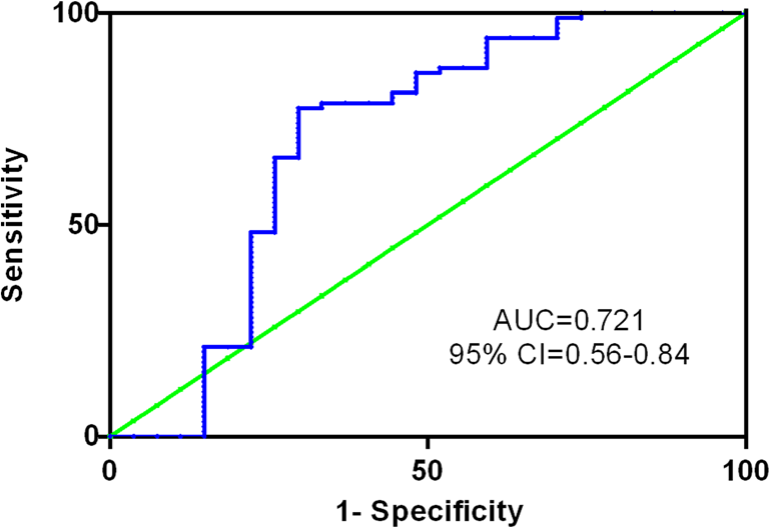
Receiver operating characteristic (ROC) curve analysis of BCL3 expression for discriminating AML patients from controls. ROC plots of BCL3 expression demonstrated the area under the curve (AUC) of 0.721 (95%CI:0.5922–0.85; *P* < 0.001) with 97.6% sensitivity and 55.6% specificity

The discriminative capacity of BCL3 was revealed by ROC curve analysis, and the results indicated that BCL3 with and AUC value of 0.721 (95% CI: 0.56–0.84) might serve as a potential biomarker for distinguishing between AML and controls (*P* < 0.001, Fig. 2).

### Association of *BCL3* Expression with Clinical Characteristics in AML

The whole-cohort of AML patients were divided into two groups (BCL3^high^ and BCL3^low^) by median value. The correlation between *BCL3* expression and clinical characteristics was present in Table 1. There were no significant differences in sex, age, white blood cells, hemoglobin, bone marrow blasts, peripheral blood blasts, karyotypes and karyotypic classifications between two groups (BCL3^high^ vs BCL3^low^, all *P* > 0.05). AML patients in the BCL3^high^ group had an increased number of platelet (median 66.33, range 10–586) compared that in the BCL3^low^ group (median 41, range 10–179) (*P* = 0.017). Significant differences were observed in the distribution of FAB subtypes between BCL3^high^ and BCL3^low^ groups. Among the FAB subtypes, the incidence of low *BCL3* expression in M2 subtype was significantly higher than in the other subtypes M1/M2/M4/M5/M6/M7 (30/17, *P* = 0.006), whereas the frequency of high *BCL3* expression in myelomonocytic/monocytic leukemia subtypes is mildly higher than leukemia with other cell types (26/50, *P* = 0.064). Among WHO subtypes, the frequency of low *BCL3* expression in AML patients with t(8;21) was the highest (23/27, *P* = 0.0047), and in AML patients without chromosomal abnormalities was the lowest (4/46, *P* = 0.006). Moreover, patients with low *BCL3* expression harbored a higher frequency of C-kit mutations (9/41, 81.8%, *P* = 0.0232) and lower frequency of FLT3-ITD mutations (1/49, 16.7%, *P* = 0.0486).

### Prognostic Value of BCL3 Expression in AML

Follow-up data was obtained in 100 AML patients. Due to independent disease entity, acute promyelocytic (M3) leukemia was excluded from the analysis. Among whole-corhort AML, no significant differences were observed in complete remission rate after the induction treatment (CR, *P* = 0.499) between two groups.

**Fig. 3:**
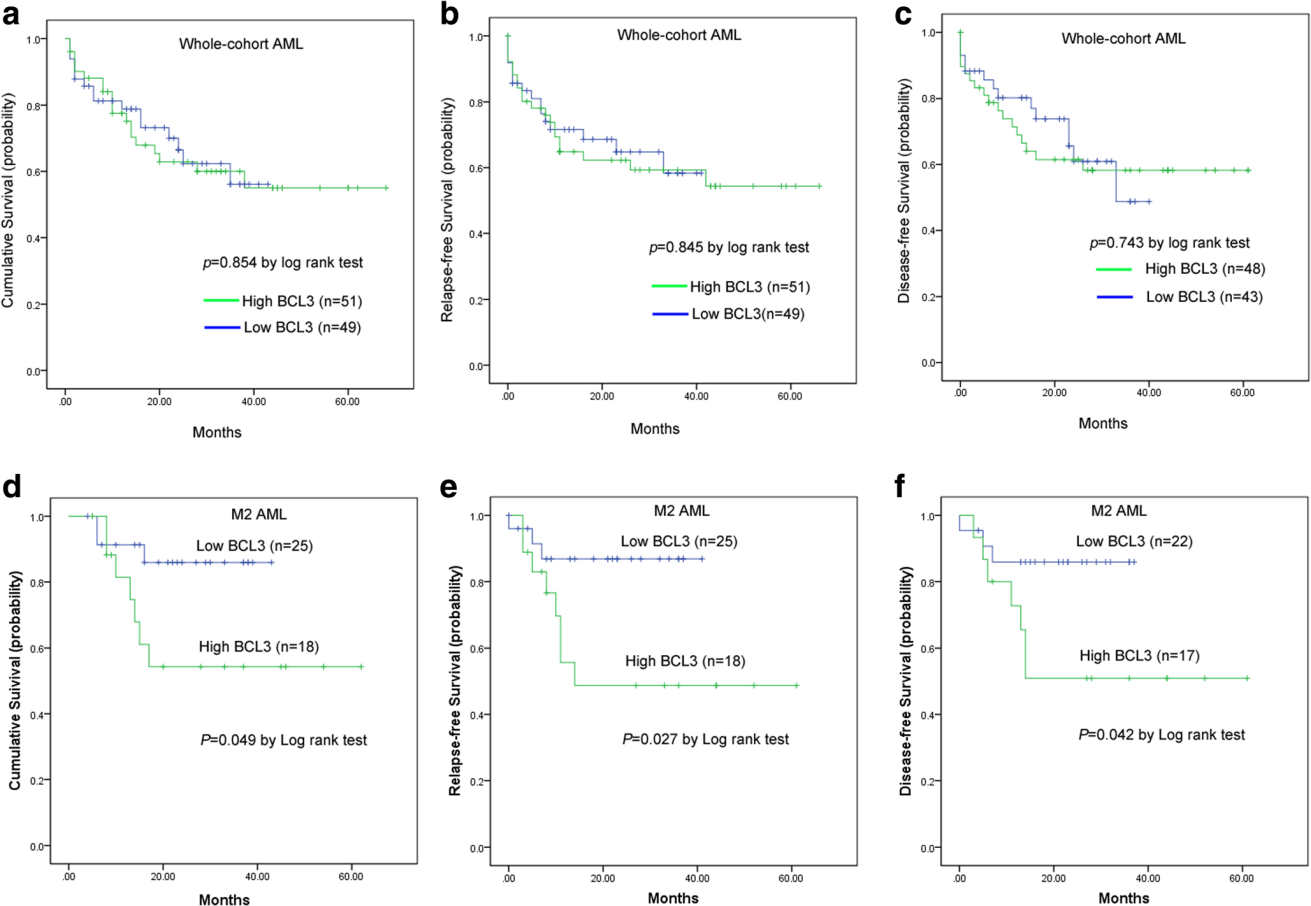
The impact of BCL3 expression on survival of AML patients. Survival analysis was performed by Kaplan-Meier methods. **a** Overall survival, OS. **b** Relapse-free survival, RFS. **c** Disease-free survival, DFS

**Fig. 4:**
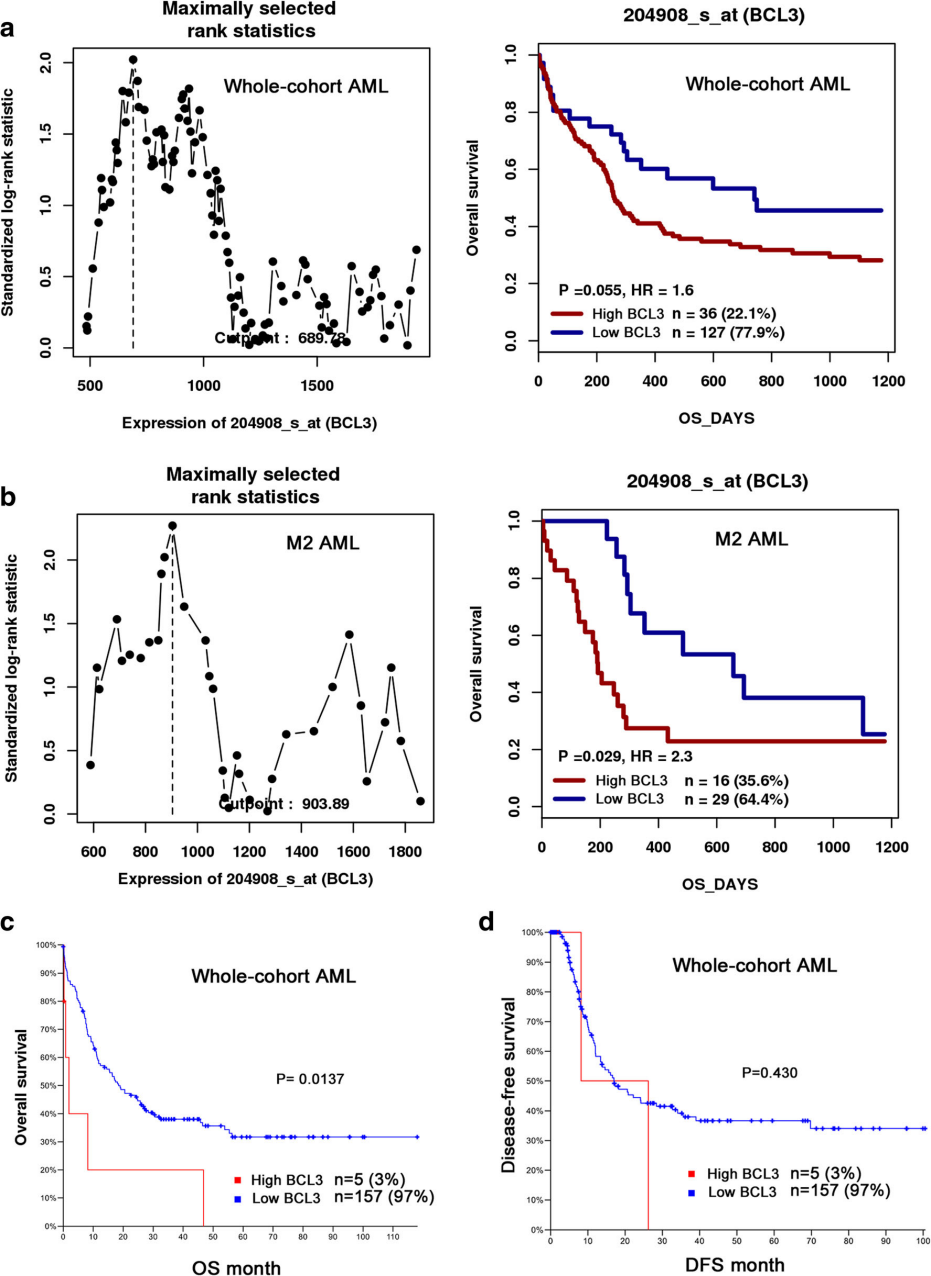
The effect of BCL3 expression on overall survival in AML patients by bioinformatics analysis. **a** and **b** survival analysis was performed by using the online web tool GenomicScape (http://genomicscape.com/microarray/survival.php). A cohort of 163 AML patients including 45 M2 subtype was obtained from Gene Expression Omnibus data (http://www.ncbi.nlm.nih.gov/geo/; accession number GSE12417). **c** and **d** overall survival and disease-free survival were conducted by the website cBioPortal (http://www.cbioportal.org/index.do)

In parallel, Survival analysis was performed in 100 non-APL patients with follow-up data ranged from 1 to 68 months (median 19.5 months). Kaplan-Meier analysis revealed that there was no significant difference in overall survival (OS), relapse-free survival (RFS) as well as disease-free survival (DFS) between BCL3^low^ and BCL3^high^ groups in whole-corhort AML patients (*P* = 0.854, 0.845 and 0.743, (Figure 3a-c). The distribution frequencies of BCL3^low^ and BCL3^high^ in M2 subtype of AML are statistically different, which suggested an association of BCL3 expression with the type of AML. Among patients with M2, high BCL3 expression presented that significantly shorter OS (median 16 versus 22 months, respectively, *P* = 0.049, Fig. 3d), RFS (median 11 versus 21 months, respectively, *P* = 0.027, Fig. 3e) and DFS (median 14 versus 21.5 months, respectively, *P* = 0.042, Fig. 3f) time compared with low BCL3 expression. Moreover, multivariate analyses were further conducted to determine the prognostic value of BCL3 expression on OS among M2 AML patients. To further validate the prognostic value of BCL3 expression in M2 AML, we focused on a cohort of M2-AML patients from the GEO data (Accession number GSE12417). Using the online web tool Genomicscape, BCL3^low^ patients showed a longer OS than BCL3^high^ among AML (HR = 1.6, *P* = 0.055) and M2 subtype AML patients (HR = 2.3, *P* = 0.029, Fig. 4a, b). Survival analysis was also conducted using the online software cBioportal, patients with BCL3 upregulation presented significantly shorter OS time (median 1.9 month) than those without BCL3 upregulation (median18.5 month) in whole-cohort AML (*P* = 0.0137, Fig. 4c, d).

## Discussion

In the present study, we for the first time identified that BCL3 was differentially expressed in de novo pediatric AML patients compared to healthy controls. BCL3 was statistically significant lower expression levels in bone marrow mononuclear cells of pediatric AML patients, and confirmed using ROC curve analysis revealing that BCL3 was a promising candidate biomarker for pediatric AML at prognosis. This is consistent with findings of previously studies reporting dysregulation of BCL3 expression in T/B cell leukemia [16–18], Hodgkin lymphoma [19, 20], T/B cell lymphoma [21, 22]. Apart from hematology malignancies, BCL3 has been shown to be participated in progression of diverse solid tumors, such as breast cancer [15], renal-cell carcinoma [13], non-small-cell lung cancer [12], cervical cancer [11], colorectal cancer [23]. Although dysregulation and oncogenetic roles of BCL3 in hematology malignancies are well established, the diagnostic and prognostic values of this gene are still to be evaluated.

In the correlation analysis of BCL3 expression with clinic-pathologic features, the M2 subtype of acute myeloblastic leukemia with maturation had a significantly higher frequency of low BCL3 expression than other subtypes. Furthermore, a mildly increased incidence of high BCL3 expression was observed in M4/M5 subtypes of acute myelomonocytic/monocytic leukemia. Consistent with our findings, two groups reported that BCL3 deficient myeloid progenitors demonstrated an enhanced capacity to differentiate into granulocytes following G-CFS stimulation, indicating that BCL3 played an important role in myelopoiesis/granulopoiesis [24, 25]. Coincidentally, Strauss L et al. stated that BCL3 was involved in granulo-and monocytopoiesis in tumor microenvironment [26]. In addition, our results demonstrated that patients with low BCL3 expression had a tendency of higher proportion of peripheral blood blasts. Taken together, these findings suggested a crucial role of BCL3 in regulation of myeloid differentiation. Andrew S et al. showed that BCL3 could affect the count and function of human platelet via mTOR-dependent pathway [27]. Consistently, our observation also revealed a relationship between BCL3 expression and platelet count, which is associated with overall survival, might explain its relation to disease prognosis.

In addition to the diagnostic value of BCL3 in cancers, prognostic value of BCL3 remains controversial in human cancers. Elevated expression of BCL3 acted as unfavorable prognostic indicator has been reported in chronic lymphocytic leukemia, non-Hodgkin’s lymphoma, colorectal cancer, breast cancer [14, 20, 23, 15, 12]. Additionally, subcellualar location of BCL3 also is related to prognosis. Karunakar et al. stated there was a difference in protein level and subcellular location of BCL3 between neoplastic tissues and adjacent normal tissue from the same colon cancer patients, and pointed out that analysis of the subcellular localization of BCL3 could be a potential-early diagnostic marker in colon cancer [23]. Consistently, our study also observed an impact of BCL3 expression on OS among AML subtype of FAB M2. BCL3 is a well established factor for maintaining cancer cell proliferation, ant-apoptosis and survival, might explain its relation to disease prognosis.

In summary, our findings suggest that BCL3 is overexpressed and presents a poor prognosis, could be also used a potential biomarker monitoring disease surveillance of Chinese pediatric AML patients.
